# The Dynamic SecYEG Translocon

**DOI:** 10.3389/fmolb.2021.664241

**Published:** 2021-04-15

**Authors:** Julia Oswald, Robert Njenga, Ana Natriashvili, Pinku Sarmah, Hans-Georg Koch

**Affiliations:** ^1^Institute for Biochemistry and Molecular Biology, Zentrum für Biochemie und Molekulare Medizin (ZMBZ), Faculty of Medicine, Albert Ludwigs Universität Freiburg, Freiburg, Germany; ^2^Faculty of Biology, Albert Ludwigs Universität Freiburg, Freiburg, Germany

**Keywords:** SecYEG translocon, protein transport, YidC, signal recognition particle, SecA, PpiD, FtsY, stress response

## Abstract

The spatial and temporal coordination of protein transport is an essential cornerstone of the bacterial adaptation to different environmental conditions. By adjusting the protein composition of extra-cytosolic compartments, like the inner and outer membranes or the periplasmic space, protein transport mechanisms help shaping protein homeostasis in response to various metabolic cues. The universally conserved SecYEG translocon acts at the center of bacterial protein transport and mediates the translocation of newly synthesized proteins into and across the cytoplasmic membrane. The ability of the SecYEG translocon to transport an enormous variety of different substrates is in part determined by its ability to interact with multiple targeting factors, chaperones and accessory proteins. These interactions are crucial for the assisted passage of newly synthesized proteins from the cytosol into the different bacterial compartments. In this review, we summarize the current knowledge about SecYEG-mediated protein transport, primarily in the model organism *Escherichia coli*, and describe the dynamic interaction of the SecYEG translocon with its multiple partner proteins. We furthermore highlight how protein transport is regulated and explore recent developments in using the SecYEG translocon as an antimicrobial target.

## Introduction

**FIGURE 1:**
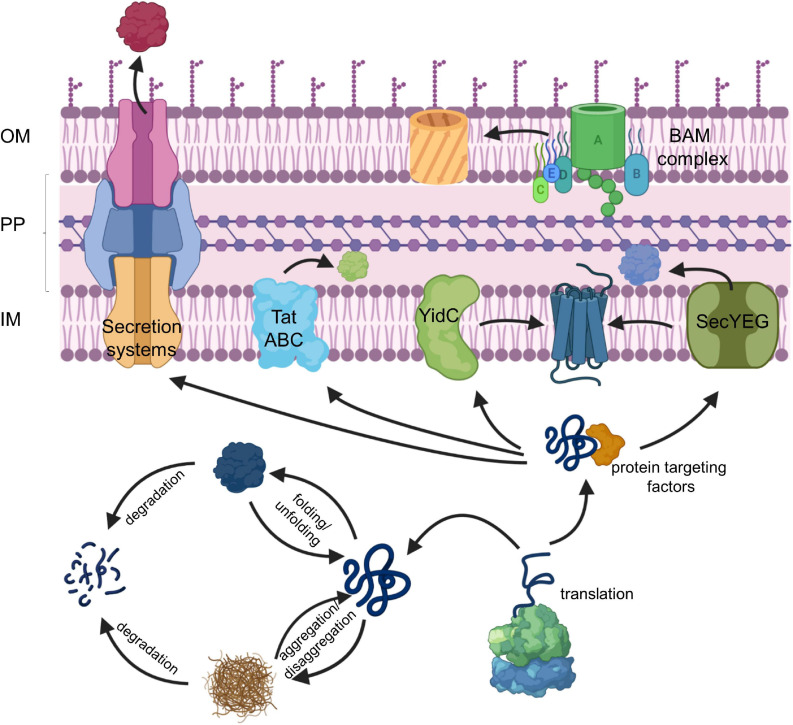
The proteostasis network in bacteria. For details see text. Secretion systems refer to the type I–IX protein secretion systems that have been identified in bacteria, although some of these secretion systems are only found in some species (Christie, 2019). IM, inner membrane; PP, periplasm; OM, outer membrane.

The dynamic control of protein synthesis, folding and degradation under different environmental conditions is essential for maintaining a functional proteome in eu- and prokaryotic cells (Mogk et al., 2011; Song et al., 2020). Protein trafficking pathways expand this proteostasis network and target proteins into subcellular compartments with specific folding conditions (Figure 1; Kudva et al., 2013; Tsirigotaki et al., 2017). Cell compartmentalization is a unifying principle in all cells and diversifies their metabolic activity by generating membrane-bordered reaction chambers. Prokaryotes lack the sophisticated intracellular organization that is usually observed in eukaryotes, but still maintain distinct compartments like the cytosol, the inner membrane, the periplasm and in Gram-negative bacteria also the outer membrane (Figure 1). Each extra-cytosolic compartment contains a dedicated protein composition which can only be maintained due to the presence of protein transport systems that export proteins out of the cytosol. The Gram-negative model organism *Escherichia coli* synthesizes approx. 4.400 different proteins^1^ and contains a predicted total number of 3–4 × 10^6^ proteins per cell, calculated based on cell volume, average protein mass and average cellular protein concentration (Milo, 2013). Ribosome profiling studies suggest that roughly one third of these proteins, accounting to approx. 1.5 × 10^6^ proteins per cell, execute their function outside of the cytosol (Li et al., 2014). The *STEPdb* databank of subcellular topologies of *E. coli* polypeptides^2^ lists approx. 1,000 different inner membrane proteins, approx. 400 periplasmic proteins and approx. 160 outer membrane proteins (OMPs) (Loos et al., 2019), all of which have in common the requirement for dedicated protein transport systems. N-terminal, cleavable signal sequences in secretory proteins and non-cleavable signal anchor sequences in inner membrane proteins provide the means to identify those proteins that have to be exported (Pugsley, 1990; von Heijne, 1994; Hegde and Bernstein, 2006; Steinberg et al., 2018).

The majority of exported proteins engage the SecYEG translocon, a universally conserved protein transport channel that resides in the inner bacterial membrane and facilitates the insertion of membrane proteins into the inner membrane as well as the translocation of proteins across the inner membrane into the periplasm (Figure 1; Kudva et al., 2013; Denks et al., 2014). The heterotrimeric SecYEG translocon consists of SecY, SecE, and SecG as core proteins, but constitutes only a passive and sealed pore that connects the cytoplasm to the periplasm and the lipid phase of the membrane. For being active in protein transport, the SecYEG translocon depends on the coordinated interaction with multiple partner proteins that select potential SecYEG substrates (Lill et al., 1990; van der Does et al., 1996; Angelini et al., 2005), provide the driving force for protein transport (Tsukazaki et al., 2011; Knyazev et al., 2018), coordinate substrate release from the SecYEG channel (Beck et al., 2001; Houben et al., 2004; Sachelaru et al., 2017) and communicate with components of the proteostasis network (Kihara et al., 1996; Schäfer et al., 1999; Jauss et al., 2019). The SecYEG translocon also cooperates with additional protein transport systems (Figure 1), like the YidC insertase (Scotti et al., 2000; Sachelaru et al., 2015, 2017; Dalbey et al., 2017; Petriman et al., 2018), the Tat transport machinery (Keller et al., 2012; Kudva et al., 2013; Tooke et al., 2017) and the Bam complex (Wang et al., 2016; Alvira et al., 2020), which inserts β-barrel proteins into the outer membrane. Additional partner proteins of the SecYEG translocon have been recently identified by proteomic approaches (Chorev et al., 2018; Carlson et al., 2019; Jauss et al., 2019), further highlighting the dynamic nature of the SecYEG translocon, which is probably the basis for its ability to transport a large variety of highly different substrates.

## Targeting the Secyeg Translocon

**FIGURE 2:**
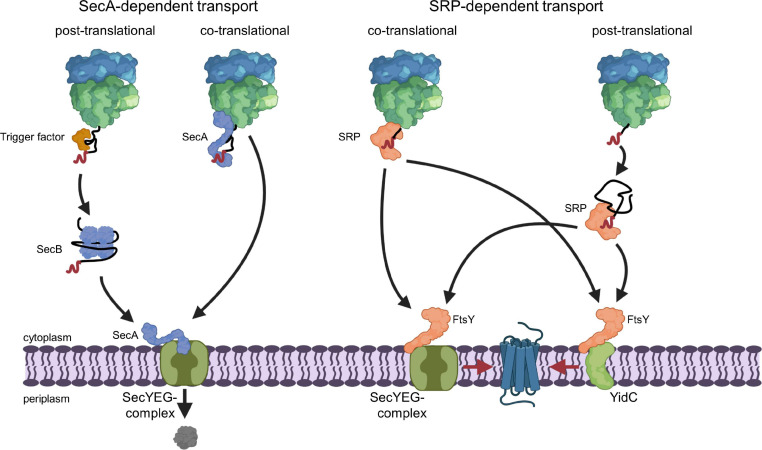
SecA- and SRP-dependent protein targeting in bacteria. The SecA- and SRP-dependent protein targeting pathways constitute the two main protein targeting pathways in bacteria and both can operate in a co- or post-translational mode. However, post-translational targeting of secretory proteins by SecA and co-translational targeting of membrane proteins by SRP are the preferred modes. Substrates of the post-translational SecA pathway are kept in a translocation competent state by chaperones, like the ribosome-bound TF or the cytosolic SecB. SecA serves as receptor for signal sequences (shown in red) of secretory proteins and is bound to the SecYEG translocon, which serves as main protein transport channel in bacteria. Repetitive ATP hydrolysis cycles by SecA allows for the translocation of the polypeptide across the SecY channel. SecA can also associate with the ribosome and target potential substrates co-translationally to the SecYEG translocon. The subsequent ATP-dependent translocation likely occurs then post-translationally, i.e., after the substrate is released from the ribosome. SRP binds with high affinity to translating ribosomes and traps the signal anchor sequence of a membrane protein when it emerges from the ribosomal peptide tunnel. SRP then delivers the translating ribosome (ribosome-associated nascent chain, RNC) to the SRP receptor FtsY. FtsY serves as SecYEG-bound receptor for nascent membrane proteins and engages similar binding sites as SecA on the SecYEG translocon. After SRP-FtsY contact, the translating ribosome docks onto the SecYEG translocon and ongoing translation inserts the protein into the lipid phase. FtsY can also associate with the YidC insertase and SRP can deliver less complex membrane proteins co-translationally to the YidC insertase for insertion. Small membrane proteins (<50 amino acids) and likely tail-anchored membrane proteins are post-translationally bound by SRP and targeted to SecYEG or YidC only after they have been released from the ribosome. This post-translational insertion by SRP is likely initiated by a so far largely uncharacterized mRNA-targeting step (Steinberg et al., 2020), which is not depicted in this cartoon.

The selective recognition of SecYEG substrates is achieved by two protein targeting systems that operate in parallel in bacterial cells (Koch et al., 2003; Rapoport, 2007; Driessen and Nouwen, 2008; Kudva et al., 2013; Smets et al., 2019). SecA-dependent protein targeting primarily acts on secretory proteins that contain a cleavable N-terminal signal sequence and this pathway is generally described as post-translational event (Figure 2). In contrast, inner membrane proteins with non-cleavable signal anchor sequences engage the signal recognition particle (SRP)-dependent targeting pathway, which operates primarily co-translationally and involves the ribosome-bound SRP (Pool et al., 2002; Gu et al., 2003; Halic et al., 2004; Schaffitzel et al., 2006) and the SecYEG-bound SRP receptor FtsY (Angelini et al., 2005, 2006; Kuhn et al., 2015; Draycheva et al., 2016; Steinberg et al., 2018; Figure 2). The SRP pathway can deliver membrane proteins also to the YidC insertase (Welte et al., 2012; Dalbey et al., 2017; McDowell et al., 2021), which can insert membrane proteins independently of SecYEG but also cooperates with the SecYEG translocon (Houben et al., 2000; Scotti et al., 2000; Serek et al., 2004; du Plessis et al., 2006; Yuan et al., 2007; Sachelaru et al., 2015, 2017; Dalbey et al., 2017). It is important to emphasize that the classification into post-translational targeting by SecA and co-translational targeting by SRP does not apply to all substrates. A co-translational targeting by SecA has been observed for the inner membrane protein RodZ, which contains a large cytosolic domain preceding its single transmembrane domain (Rawat et al., 2015; Wang et al., 2017), and for the periplasmic maltose binding protein MBP (Huber et al., 2017). This is in line with the ability of SecA to interact with translating and non-translating ribosomes (Eisner et al., 2003; Karamyshev and Johnson, 2005; Huber et al., 2011; Knupffer et al., 2019; Origi et al., 2019; Wang S. et al., 2019). On the other hand, a post-translational interaction of SRP has been shown for the small bacterial membrane proteins YohP and YkgR (Steinberg et al., 2020) and for the tail-anchored proteins DjlC, Flk, and SciP (Pross et al., 2016; Peschke et al., 2018).

### Targeting by SecA

**FIGURE 3:**
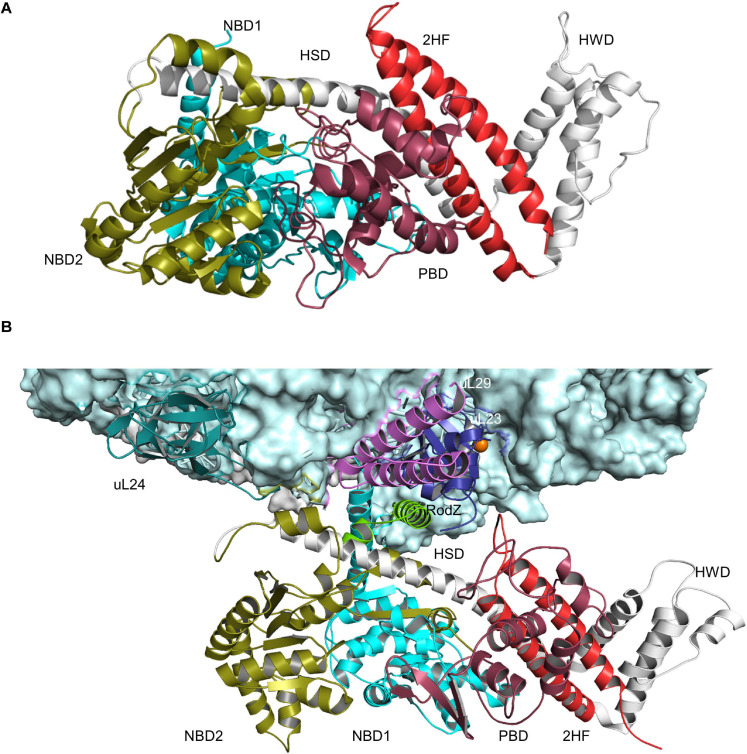
Structures of SecA and SecA bound to the ribosome. **(A)** Structure of *B. subtilis* SecA (PDB 5EUL), showing its multiple domains. The two nucleotide-binding domains NBD1 and NBD2 are shown in cyan and in olive, respectively. The peptide-binding domain (PBD) is shown in raspberry-red, the helical wing domain (HWD) and the helical scaffold domain (HSD) in gray and the two-helix finger (2HF) in red. **(B)** Structure of *E. coli* SecA bound to a translating ribosome (PDB 6S0K). The 50S ribosomal subunit is shown in light-blue and the nascent RodZ chain in green. Ribosomal proteins that are in contact with SecA [uL23 (blue), uL29 (pink), and uL24 (green)] and the different domains of SecA are labeled and shown in the same color-code as in **(A)**.

The ATPase SecA is a multi-domain protein of 102 kDa that is found exclusively in bacteria and chloroplasts (Pohlschroder et al., 1997; Figure 3A). In *E. coli* it is present in about 2,000–5,000 copies per cell (Kudva et al., 2013; Smets et al., 2019) and therefore much more abundant than the SecYEG complex, which exists in about 500 copies (Kudva et al., 2013). SecA binds with high-affinity to the cytosolic loops of SecY (Douville et al., 1995; Mori and Ito, 2006; Kuhn et al., 2011) and to negatively charged phospholipids (Lill et al., 1990; Gold et al., 2010; Koch et al., 2016, 2019). In addition, a fraction of SecA is located in the cytosol (Chun and Randall, 1994; Hoffschulte et al., 1994), where it can exist as dimer (Woodbury et al., 2002; Banerjee et al., 2017a). The oligomeric state of membrane-bound SecA is controversially discussed. Liposome studies indicate that only the SecA monomer binds to phospholipids (Roussel and White, 2020), but a SecA dimer is functional in protein translocation (de Keyzer et al., 2005) and can function as receptor for preproteins (Gouridis et al., 2013). It has been suggested that one protomer is required for docking onto the SecYEG complex, while the second copy is involved in the downstream translocation upon ATP-dependent dissociation of the dimer (Or et al., 2002; Gouridis et al., 2013).

SecYEG-bound SecA primarily recognizes its substrates after they have been released from the ribosome (Randall, 1983; Hartl et al., 1990; Swidersky et al., 1990; Chun and Randall, 1994; Fekkes et al., 1998). N-terminal signal sequences are bound via a shallow groove within the preprotein-binding domain (PBD) of SecA, also called preprotein cross-linking domain (PPXD) (Gelis et al., 2007; Grady et al., 2012). The PBD domain is located close to the two nucleotide binding domains (NBD1 and NBD2) and dynamic movements within the PBD link substrate recognition to ATP binding and hydrolysis (Karamanou et al., 2007; Gouridis et al., 2013; Figure 3A). Although signal sequences are probably the most important determinants for SecA-dependent targeting (Hegde and Bernstein, 2006), additional sequences within the mature domain of a secretory protein can also contribute to the specificity of the targeting reaction (Chatzi et al., 2017). Binding of SecA to sequences within the mature domain might be in particular important for keeping substrates in a translocation competent state, e.g., largely unfolded. Translocation competence is furthermore supported by chaperones like Trigger factor (TF) (Saio et al., 2014, 2018; Can et al., 2017; De Geyter et al., 2020) or SecB (Bechtluft et al., 2010; Huang et al., 2016; Figure 2). Due to its high affinity to ribosomes and its ability to bind to the ribosomal protein uL23 (Kramer et al., 2002), TF is one of the first contacts of the emerging nascent chain (Deuerling et al., 1999; Bornemann et al., 2014). Different to SecA, TF does not specifically bind to signal sequence-containing proteins but also binds to cytosolic proteins, although β-barrel OMPs appear to be the preferred target (Teter et al., 1999; Oh et al., 2011). It has been shown that protein translocation of some substrates is accelerated upon TF deletion and it was suggested that this reflects prolonged contact between TF and these outer membrane substrates (Lee and Bernstein, 2002). TF can also interact with SecB and the SecYEG-bound SecA, which probably helps to connect protein folding and protein transport (De Geyter et al., 2020). SecB is present in proteobacteria only and like TF not essential (Deuerling et al., 2003; Crane and Randall, 2017). It forms a tetramer with surface-exposed hydrophobic areas, which are involved in substrate binding (Knoblauch et al., 1999). SecB binds only to a small number of secretory proteins and releases its substrates upon binding to the C-terminus of SecA (Baars et al., 2006; Crane et al., 2006; Castanie-Cornet et al., 2014).

In addition to this post-translational substrate recognition, SecA can bind to its substrates also co-translationally (Eisner et al., 2003; Karamyshev and Johnson, 2005; Huber et al., 2011, 2017; Figure 2). This was observed for secretory proteins, like MBP (Chun and Randall, 1994; Huber et al., 2017), but also for the membrane protein RodZ (Rawat et al., 2015; Wang et al., 2017). SecA binds to the ribosome close to the ribosomal tunnel exit, which is formed by the ribosomal proteins uL23, uL24, and uL29 (Huber et al., 2011; Knupffer et al., 2019; Wang S. et al., 2019; Figure 3B). This is also the binding site for SRP and for many ribosome-associated chaperones and processing factors (Kramer et al., 2002, 2009; Denks et al., 2017; Knupffer et al., 2019). Importantly, it is the N-terminus of SecA that interacts with both the ribosome and with SecYEG or phospholipids (Knupffer et al., 2019; Origi et al., 2019) and thus SecA binding to ribosomes or to SecYEG appears to be mutual exclusive. This suggests that co-translational targeting by SecA is followed by a post-translational translocation across the SecYEG translocon. This assumption is also in line with the observation that SecA and ribosomes use almost identical binding sites on SecY (Prinz et al., 2000; Mori and Ito, 2006; Kuhn et al., 2011; Banerjee et al., 2017b) and that SecA and ribosomes compete for SecYEG binding (Wu et al., 2012).

### Targeting by the SRP Pathway

**FIGURE 4:**
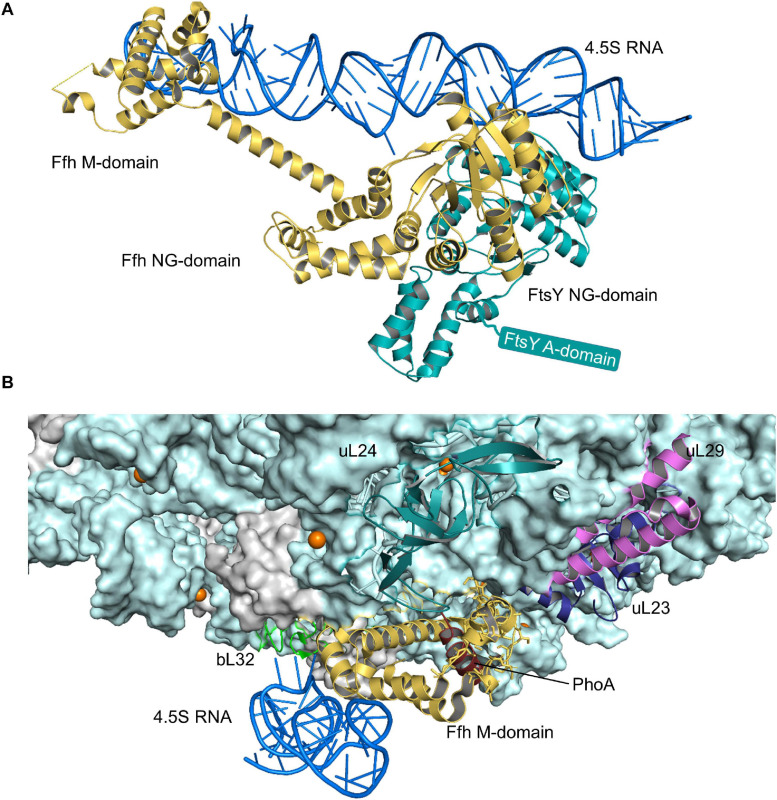
Structures of the SRP-FtsY-complex and the SRP-ribosome complex. **(A)** Structure of the *E. coli* SRP-FtsY complex (PDB 2XXA) (Ataide et al., 2011). Ffh, the protein component of the bacterial SRP is shown in yellow and the 4.5S RNA in dark-blue. The domains of Ffh are indicated. The NG-domain of FtsY is shown in green; the structure of the N-terminal A-domain of FtsY has not been solved yet and is shown as green box. **(B)** Structure of an SRP-RNC complex (PDB 5GAH). The 50S ribosomal subunit is shown in light-blue and the ribosomal proteins that provide the contact site for SRP are indicated, uL23 (blue), uL29 (pink), uL24 (green), and bL32 (light-green). Ffh is shown in yellow and the 4.5S RNA in dark-blue. The nascent PhoA chain is shown in dark red.

The SRP pathway is a universally conserved targeting system that bacteria primarily use for inner membrane proteins (Figure 2) (Ulbrandt et al., 1997; de Gier et al., 1998; Valent et al., 1998; Cristobal et al., 1999; Koch et al., 1999, 2003; Koch and Muller, 2000). In *E. coli*, SRP consists of the protein Ffh and the 4.5S RNA (Figure 4A) and thus represents a basic version of the eukaryotic SRP, which consists of six protein subunits bound to the 7SL RNA (Koch et al., 2003). Still, the bacterial SRP and its receptor FtsY are sufficient to support protein targeting to mammalian endosomal membranes (Powers and Walter, 1997). The SRP pathway in bacteria not only targets the SecYEG translocon, but also the YidC insertase (Welte et al., 2012; Petriman et al., 2018), which inserts less-complex membrane proteins (Samuelson et al., 2000; Dalbey et al., 2017). Ffh and FtsY share a homologous NG domain with highly similar architecture and amino acid sequence (Freymann et al., 1997; Montoya et al., 1997). The respective N-domains form a four-helix bundle that is followed by the Ras-like GTPase domain (G-domain) (Figure 4A). The NG-domain of Ffh is C-terminally continued by the M-domain, which forms a flexible groove that is able to accommodate signal anchor sequences of different lengths and hydrophobicities. This flexibility explains why the bacterial SRP recognizes the hydrophobic signal anchor sequences of basically all inner membrane proteins and also the signal sequences of some secretory proteins and amphipathic helices of integral and membrane-associated proteins (Beha et al., 2003; Huber et al., 2005; Maier et al., 2008; Lim et al., 2013; Schibich et al., 2016). Substrate recognition by SRP is a multi-step process that is initiated by SRP binding to the ribosome, where it contacts primarily uL23, uL29, and the 23S rRNA close to the tunnel exit (Halic et al., 2006a, b; Schaffitzel et al., 2006; Figure 4B). SRP binds to vacant ribosomes with high affinity (*K*_*d*_ 50–60 nM) (Bornemann et al., 2008; Holtkamp et al., 2012) and the flexible C-terminus of Ffh protrudes into the ribosomal tunnel where it contacts the intra-tunnel loop of uL23 (Jomaa et al., 2016, 2017; Denks et al., 2017). This scanning mode allows SRP to screen ribosomes for potential substrates. When translation is initiated and the nascent chain reaches a length of approx. 25 amino acids, SRP is displaced from the intra-tunnel loop, which now contacts the nascent chain (Denks et al., 2017). However, SRP maintains contact to the surface-exposed domain of uL23 and this anticipatory or stand-by mode further increases the affinity (*K*_*d*_ 1 nM) and likely orients the M-domain for binding to the signal anchor sequence. When the nascent chain reaches a length of approx. 45–50 amino acids and the signal anchor sequence is exposed to the outside of the ribosome, SRP forms a stable complex with the ribosome-associated nascent chain (RNC) (*K*_*d*_ ≤ 1 nM) (Holtkamp et al., 2012; Schibich et al., 2016; Denks et al., 2017). The SRP-RNC complex is then targeted to the SRP receptor FtsY. Although some initial studies proposed that the SRP-RNC complex interacts with FtsY already in the cytosol (Shan et al., 2007; Saraogi et al., 2014), FtsY in Gram-positive and Gram-negative bacteria is almost exclusively membrane-bound (Mircheva et al., 2009).

**FIGURE 5:**
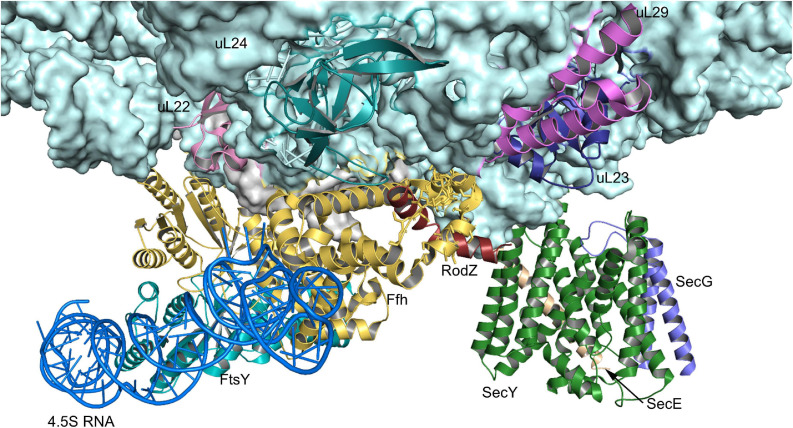
Structure of the quaternary RNC-SRP-FtsY-SecYEG complex. Structure of the quaternary complex (PDB 5NCO), depicting an early state of co-translational protein insertion. The subunits SecY, SecE and SecG of the SecYEG translocon are indicated by green, orange and blue color, respectively. The color code of the FtsY-SRP complex is as in Figure 4 and the nascent PhoA is shown in dark-red. Please note that in this structure, the SecYEG translocon is only tentatively fitted and would have to tilt by ∼20° to be accommodated within the membrane (Jomaa et al., 2017).

Membrane binding of FtsY is mediated by the A-domain, which precedes the NG-domain (Figure 4A), and by a membrane-targeting sequence at the interface of the A- and NG-domains (de Leeuw et al., 2000; Parlitz et al., 2007; Weiche et al., 2008; Braig et al., 2009; Erez et al., 2010; Kuhn et al., 2011). The A-domain is highly variable in length and sequence and so far no structural information is available, suggesting intrinsic flexibility (Montoya et al., 1997). The A-domain is not essential for protein targeting in *E. coli* (Eitan and Bibi, 2004), which is explained by the presence of additional binding sites for SecY and phospholipids in the N-domain of FtsY (Parlitz et al., 2007; Weiche et al., 2008; Braig et al., 2009; Erez et al., 2010; Kuhn et al., 2011). However, the A-domain is important for increasing the fidelity of the targeting reaction because it shields the SRP binding site when FtsY is not in contact with the SecYEG complex (Draycheva et al., 2016; Lakomek et al., 2016) and it thus prevents futile SRP-FtsY interactions. Binding of SRP-RNCs to the FtsY-SecYEG complex generates a transient quaternary complex (Kuhn et al., 2015; Jomaa et al., 2017; Draycheva et al., 2018; Figure 5). Subsequent movements of SRP expose the SecY binding site on the ribosome (Halic et al., 2006b) and simultaneous movements of FtsY expose the ribosome binding site on SecY (Halic et al., 2006b; Kuhn et al., 2015). This then allows for the docking of the RNC onto the SecYEG translocon and subsequent GTP hydrolysis by the FtsY-SRP complex (Egea et al., 2004; Focia et al., 2004; Saraogi et al., 2014). GTP-hydrolysis induces the dissociation of the FtsY-SRP complex and allows for the next round of targeting (Egea et al., 2004; Shan et al., 2004; Akopian et al., 2013a).

Importantly, the SecA and SRP pathways have several features in common: (1) SecA and SRP engage the same docking site on the ribosome and both protrude into the ribosomal tunnel (Denks et al., 2017; Knupffer et al., 2019; Wang S. et al., 2019). (2) FtsY and SecA are activated upon binding to anionic phospholipids and SecY (Mircheva et al., 2009; Kuhn et al., 2011; Stjepanovic et al., 2011; Draycheva et al., 2016; Koch et al., 2016). (3) FtsY, SecA and the ribosome use largely identical binding sites on SecY (Mori and Ito, 2006; Kuhn et al., 2011, 2015). A computational approach for investigating the early evolutionary history of protein transport systems indicates that the SRP/FtsY targeting pathway is the most ancient protein delivery system that probably even existed before the last universal common ancestor (LUCA) (Harris and Goldman, 2021). As protein transport is faster than translation (Pugsley, 1990; Rodnina and Wintermeyer, 2016), the evolution of a second targeting system in fast growing bacteria probably ensures that secretory proteins are kept in a translocation-competent state, when the limited number of SecYEG translocons are co-translationally engaged by SRP-substrates.

Finally, translation-independent membrane localization of some mRNAs encoding for membrane proteins has been observed in bacteria (Nevo-Dinur et al., 2011; Kannaiah and Amster-Choder, 2014; Kannaiah et al., 2019). One example is the small membrane protein YohP, which consists of just 27 amino acids and is predicted to be involved in the bacterial stress response (Hemm et al., 2010). The *yohP* mRNA was found to be almost exclusively membrane localized, but membrane insertion of the YohP protein by either the SecYEG complex or YidC still required SRP and FtsY (Steinberg et al., 2020). SRP contacts YohP post-translationally both *in vivo* and *in vitro* (Steinberg et al., 2020), questioning the paradigm that SRP has to be ribosome-bound for substrate recognition. For small membrane proteins, the post-translational recognition by SRP can be easily explained by the fact that they are already released from the peptidyl transferase domain of the ribosome before they are sufficiently exposed on the ribosomal surface for co-translational SRP recognition. Considering the rapidly increasing number of small membrane proteins discovered in bacteria (Storz et al., 2014; Weaver et al., 2019), the post-translational targeting by SRP could be as abundant as the co-translational targeting and might also be executed for C-tail anchored membrane proteins in bacteria (Abell et al., 2004; Pross et al., 2016; Peschke et al., 2018; Figure 2).

## The Secyeg Complex in the Resting and Active State

**FIGURE 6:**
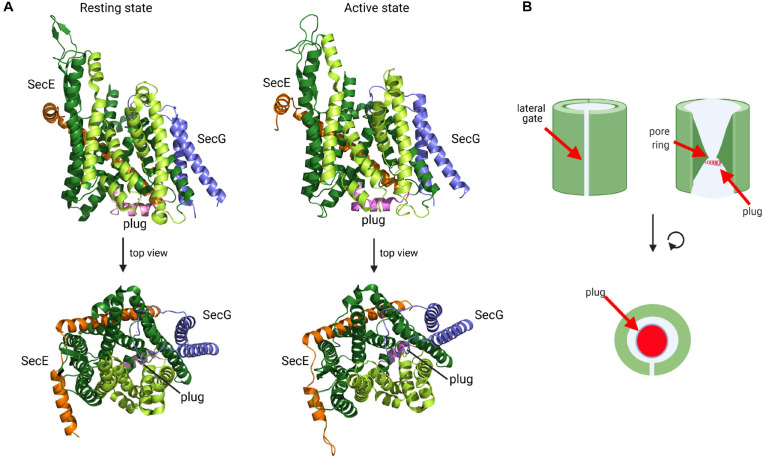
Structure of the SecYEG translocon in its resting state and active state. **(A)** Structure of *T. thermophilus* SecYEG in the resting state (PDB 5AWW) and the active state (PDB 5CH4). SecY is shown in green, SecE in orange and SecG in blue. The SecY transmembrane domains that constitute the lateral gate are shown in light green and the plug in magenta. The upper structures depict the front views of the SecYEG translocon and the lower structures the top view from the cytosol, respectively. **(B)** Schematic front view and view from the cytosol of the SecYEG translocon.

The first X-ray structure of the Sec translocon was obtained for the homologous SecYEβ complex from the archaeon *Methanococcus janaschii* and represented the resting state with a sealed pore (Van den Berg et al., 2004). In this resting conformation, which was later also obtained from other species (Li et al., 2007; Tsukazaki et al., 2008; Tanaka et al., 2015), SecY is organized in two halves formed by transmembrane helices (TMs) 1 to 5 and 6 to 10, respectively, which are connected by a loop between TM5 and 6, termed the hinge (Figure 6). In this clamshell-like structure, SecY forms two vestibules with a central constriction, called the pore ring, in the middle. The pore ring is formed by six bulky and hydrophobic isoleucine residues in *E. coli* and is sealed on the periplasmic side by a short helix (TM2a; the plug) (Figure 6B). The plug and the pore ring are important for maintaining the membrane barrier in the resting state and during translocation (Saparov et al., 2007; Park and Rapoport, 2011). This structural arrangement provided a first glimpse into how the SecY channel is able to translocate proteins across the membrane, but also to insert proteins into the membrane (Van den Berg et al., 2004). At the front of SecY, TMs 2/3, and 7/8 constitute a flexible crevice, called the lateral gate that allows access to the lipid phase (du Plessis et al., 2009; Hizlan et al., 2012; Bischoff et al., 2014; Gogala et al., 2014; Figure 6A). Cytosolically exposed loops of SecY provide the docking sites for SecA (Mori and Ito, 2006; Das and Oliver, 2011; Kuhn et al., 2011), FtsY (Angelini et al., 2005, 2006; Kuhn et al., 2011) and ribosomes (Prinz et al., 2000; Frauenfeld et al., 2011; Kuhn et al., 2011). Although these sites are not identical, they largely overlap (Kuhn et al., 2011), which indicates that SecA, FtsY and ribosomes compete for SecY binding (Wu et al., 2012; Kuhn et al., 2015). The tilted TM3 of SecE further stabilizes the hinge at the back of SecY and this appears to be crucial for its integrity because SecY is rapidly degraded by the membrane protease FtsH in the absence of SecE (Kihara et al., 1995; Lycklama a Nijeholt et al., 2013). SecG, the third subunit of the bacterial SecYEG complex, consists of two transmembrane domains, which are connected by a cytosolic loop (Figure 6). SecG is not essential for cell viability, but Δ*secG* strains of *E. coli* exhibit protein transport defects *in vivo* (Nishiyama et al., 1994, 1996).

**FIGURE 7:**
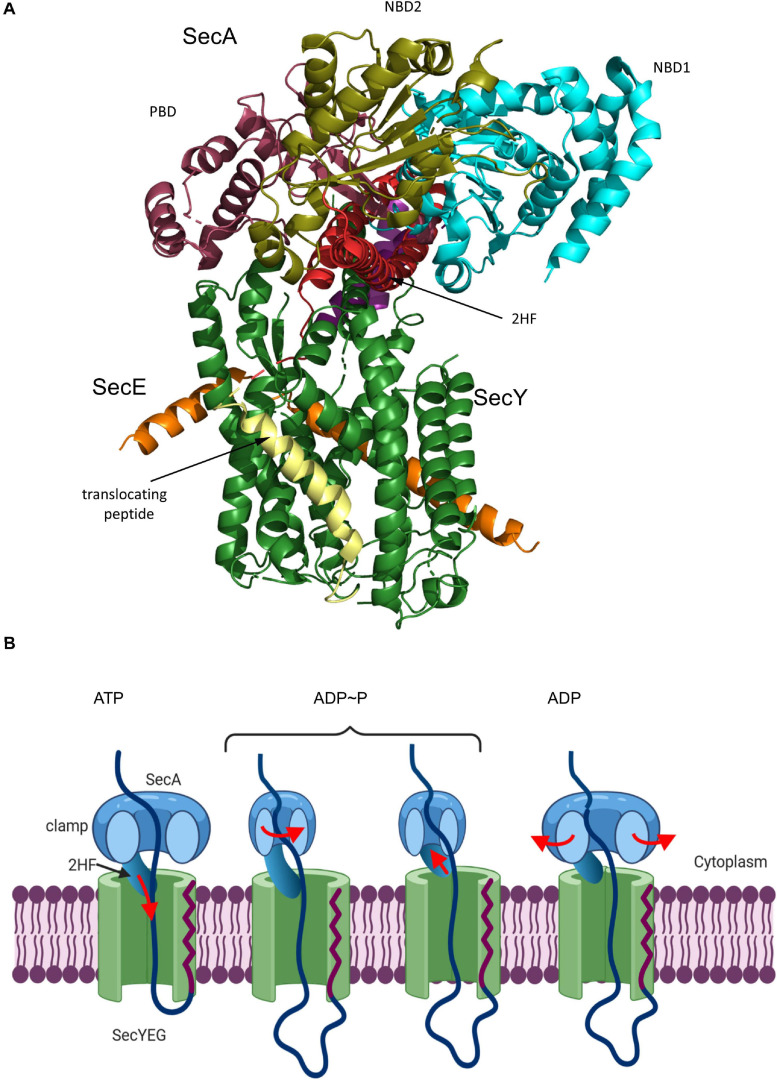
Structure of the substrate-engaged SecA-SecYEG complex and model for SecA-dependent translocation across the SecYEG-translocon. **(A)** Structure of the SecA-SecYEG complex from *B. subtilis* (PDB 5EUL). SecY and SecE are shown in green and orange, respectively, and the translocating peptide in yellow. The different domains of SecA are indicated. 2HF corresponds to the two-helix finger. **(B)** Upon ATP binding to SecA, the 2-helix-finger (2HF) inserts into the SecY channel and pushes the polypeptide into the channel. The signal sequence is depicted in red. For preventing back-sliding, the polypeptide binding domain (PBD) of SecA rotates toward the nucleotide-binding domain (NBD2) and forms a clamp that traps the polypeptide. This step likely occurs before or simultaneously with ATP-hydrolysis. Closing the clamp also leads to the retraction of the 2HF. After phosphate release, the clamp opens again and the polypeptide can slide deeper into the channel but in principle also backward. *In vivo*, backsliding at this stage could be prevented by contacts of the polypeptide to periplasmic chaperones, like Skp (Schäfer et al., 1999) or the PpiD/YfgM complex (Götzke et al., 2014; Jauss et al., 2019). In addition, the membrane potential is likely important for maintaining directionality of translocation (Driessen and Nouwen, 2008; Knyazev et al., 2018). Figure was modified after (Catipovic et al., 2019).

Activation of the SecYEG channel and subsequent protein transport requires opening of the lateral gate, expansion of the pore ring and movement of the plug (Collinson et al., 2015; Voorhees and Hegde, 2016b; Figure 6A). These movements have been documented by additional structures and a wealth of biochemical data. For the transport of secretory proteins, the SecYEG channel is activated by SecA, which serves a dual function: it acts as SecYEG bound receptor for proteins with cleavable signal sequences and provides the energy for translocation by multiple ATP-hydrolysis cycles (Douville et al., 1995; Manting et al., 1997; Tomkiewicz et al., 2006; Alami et al., 2007; Das and Oliver, 2011; Gold et al., 2013; Gouridis et al., 2013). A first structure of a SecYEG-SecA complex (Zimmer et al., 2008) revealed the insertion of the hairpin-like two-helix finger (2HF) of SecA into the cytoplasmic vestibule of SecY and a partial opening of the lateral gate. This opening is required for intercalation of the signal sequence within the lateral gate (du Plessis et al., 2009; Hizlan et al., 2012; Corey et al., 2016). This is depicted in the structure of the SecYEG-SecA complex with a covalently linked signal sequence (Li et al., 2016; Figure 7A). This structure shows that the hydrophobic segment of the signal sequence is located outside of the opened lateral gate. The segment following this hydrophobic part is trapped between TM3 and TM7 on the periplasmic part of the lateral gate and the signal sequence cleavage site is located within the periplasmic vestibule. Opening of the channel is further accompanied by movement of the plug to the back of the channel, where it resides close to SecE, validating previous cross-linking studies (Harris and Silhavy, 1999; Tam et al., 2005).

The activation of SecYEG by SecA initiates the step-wise translocation of secretory proteins across the membrane. The reconstituted SecYEG-SecA complex was shown to generate a mechanical force of about 10pN (Robson et al., 2007; Gupta et al., 2020). Consequentially, several models were proposed on how the high conformational flexibility of SecA might be used for the ATP-dependent and stepwise translocation of a preprotein across the SecYEG channel (Erlandson et al., 2008a, b; Kusters et al., 2011; Gouridis et al., 2013; Ernst et al., 2018; Fessl et al., 2018; Corey et al., 2019; Komarudin and Driessen, 2019). Central to most models is the 2HF-domain of SecA (Erlandson et al., 2008a). The 2HF was shown to insert into the cytosolic vestibule of SecY, where it resides in close proximity to the preprotein (Zimmer et al., 2008). A highly conserved tyrosine residue at the tip of the loop is essential for SecA function, but immobilizing the 2HF on the SecYEG complex does not interfere with translocation (Whitehouse et al., 2012), suggesting that even restricted movements of the 2HF are sufficient to support translocation. Latest data support a push-and-slide mechanism of protein translocation that depends on a power stroke by SecA (Catipovic et al., 2019; Catipovic and Rapoport, 2020). In this model (Figure 7B), the 2HF moves toward the SecY channel upon ATP binding, thereby pushing the polypeptide into the channel. While the 2HF retracts during ATP hydrolysis from the channel, movement of the polypeptide-binding domain of SecA toward the nucleotide-binding domain generates a clamp that fixes the polypeptide in the channel. Phosphate release from SecA is suggested to open the clamp, which allows for some passive sliding of the polypeptide until the next ATP binds and the 2HF pushes the next segment of the polypeptide into the channel. The observation that cross-linking the 2HF to the cytosolic loop C4 of SecY does not impair protein translocation (Whitehouse et al., 2012) is possibly explained by the inherent flexibility of the large C4 loop which might still allow sufficient movements of the 2HF.

The 2HF is also central to an alternative model for SecA-dependent translocation, which suggests a Brownian ratchet mechanism (Collinson, 2019). In this model, SecA regulates channel opening via the 2HF, while substrate movement across the channel occurs via Brownian movement (Allen et al., 2016, 2020). ATP hydrolysis by SecA is suggested to prevent partial folding of substrates at the SecA-SecY interface, while the partial folding on the periplasmic side would prevent back-sliding and thus impose directionality to protein translocation (Fessl et al., 2018; Corey et al., 2019).

In both models, substrate translocation is further stimulated by the proton-motif-force (PMF), which probably adds to vectorial translocation (Brundage et al., 1990; Nouwen et al., 1996; Knyazev et al., 2018). Prior to completion of translocation, the signal sequence is cleaved off by signal peptidase and the mature domain is released into the periplasm (Josefsson and Randall, 1981a, b; Paetzel et al., 2002). This latter step is likely supported by periplasmic chaperones (Schäfer et al., 1999; Furst et al., 2018; Chum et al., 2019; Mas et al., 2019) (see below).

**FIGURE 8:**
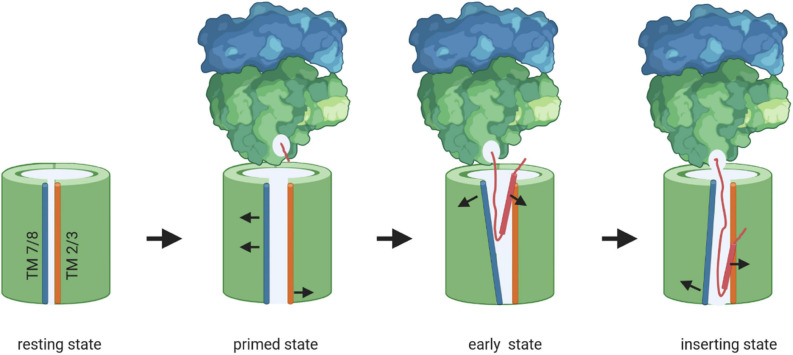
Model of membrane protein insertion via the SecYEG translocon. In the resting state of the SecYEG-translocon, the lateral gate, composed of transmembrane domains (TMs) 2/3 on one side (orange) and TMs 7/8 (blue) on the other side, is closed. Binding of the translating ribosome to the cytosolically exposed loop connecting TM 6 and 7 of SecY (C5-loop, not shown), causes the lateral gate to slightly open, which is then primed for the approaching nascent chain. The emerging nascent membrane protein (red) disrupts contacts between TM 2 and TM 7 on the cytosolic side of the membrane further, while TM 7 moves closer toward TM 3 on the periplasmic side. This creates a V-shaped crevice during the early state of insertion. This state is likely further stabilized by the two N-terminal TMs of SecE (not shown). Ongoing chain elongation positions the hydrophobic core of the signal peptide (red zylinder) at the lateral gate, where it occupies approx. the same position as TM 2 in the resting SecYEG channel, before it is released into the membrane.

Inner membrane proteins are targeted to the SecYEG translocon co-translationally as RNCs by the SRP pathway (Figure 2; Koch et al., 1999; Beck et al., 2000; Neumann-Haefelin et al., 2000; Akopian et al., 2013b; Steinberg et al., 2018). The SRP receptor FtsY docks onto the SecYEG translocon and engages largely identical binding sites as SecA and the ribosome (Angelini et al., 2005, 2006; Kuhn et al., 2011, 2015). FtsY and SecA have comparable affinities for the SecYEG translocon and are present in comparable copy numbers in *E. coli* (Douville et al., 1995; Kudva et al., 2013; Kuhn et al., 2015) and it is currently unknown how access of either FtsY or SecA to the SecYEG translocon is regulated. Importantly, only SecY-bound FtsY exposes the SRP binding site and is thus able to direct the SRP-RNC complex to the SecYEG translocon (Mircheva et al., 2009; Draycheva et al., 2016). Structural information on the isolated FtsY-SecYEG complex is not available, but Cryo-EM structures of RNCs bound to the Sec translocon in the presence and absence of SRP and its receptor have been obtained from different species (Becker et al., 2009; Frauenfeld et al., 2011; Bischoff et al., 2014; Gogala et al., 2014; Voorhees et al., 2014; Jomaa et al., 2016, 2017; Voorhees and Hegde, 2016a; Kater et al., 2019). Binding of a non-translating ribosome to the Sec translocon, primarily via the cytosolic loop C5, results in small rearrangements which slightly open the cytosolic part of the lateral gate (Voorhees et al., 2014; Figure 8). The structure of a quaternary ribosome-SRP-FtsY-SecYEG complex revealed that FtsY aligns the ribosomal tunnel exit with the SecYEG channel (Jomaa et al., 2017; Figures 5, 8). The exposure of a short nascent membrane protein further opens the lateral gate on the cytosolic side (Kater et al., 2019) and full insertion of the signal anchor sequence leads to a rotation of helices 2–5 and 10 and allows trapping of the signal anchor sequence at the lateral gate (Voorhees and Hegde, 2016a; Figure 8). Simultaneously, the plug is displaced from its position at the pore ring and the channel is open to both the trans-side and the lipid side of the membrane. TMs downstream of the signal anchor sequence can exit the Sec translocon laterally one by one or in pairs (Heinrich and Rapoport, 2003; Houben et al., 2004; Sadlish et al., 2005). Lipid partitioning of TMs is largely determined by their hydrophobicity (Hessa et al., 2007; White and von Heijne, 2008) and moderately hydrophobic TMs possibly require the interaction with a more hydrophobic second TM to enter the lipid phase (Heinrich and Rapoport, 2003). These helix-helix interactions could occur within the Sec channel (Pitonzo et al., 2009), at the channel-lipid interface (Sadlish et al., 2005; Cross and High, 2009) or even before, at the end of the ribosomal tunnel (Tu et al., 2014; Holtkamp et al., 2015; Nilsson et al., 2015). Lateral release of transmembrane domains out of the SecY channel is further facilitated by YidC (Beck et al., 2001; Houben et al., 2002), which associates with the lateral gate of SecY to form a tetrameric protein channel (Sachelaru et al., 2015, 2017).

Although there are some variations in the translocon structure when activated by SecA or the ribosome, the step-wise channel opening during post-translational translocation or co-translational insertion appears to be a conserved feature of the Sec translocon and is in line with multiple biochemical studies (du Plessis et al., 2009; Bonardi et al., 2011; Hizlan et al., 2012; Knyazev et al., 2013, 2014; Ge et al., 2014; Mercier et al., 2020). It is, however, currently unknown how channel opening and transport across the SecYEG translocon works for membrane proteins that are co-translationally targeted by SecA, like RodZ (Rawat et al., 2015; Wang et al., 2017; Figure 3). A simultaneous binding of SecA and the ribosome to SecY appears unlikely, considering that both engage overlapping binding sites on SecY (Kuhn et al., 2011). One possibility is that SecA starts inserting RodZ only after it is released from the ribosome. In this case, only targeting would occur co-translationally, while the actual insertion would be post-translationally. A similar situation is encountered during co-translational insertion of membrane proteins with large periplasmic loops, because their translocation requires SecA (Neumann-Haefelin et al., 2000; Deitermann et al., 2005). How the access of SecA to these loops during co-translational insertion is coordinated is currently unknown. Finally, how the SecYEG translocon handles small membrane proteins that are post-translationally targeted by SRP (Steinberg et al., 2020; Figure 2), i.e., when neither the ribosome nor SecA are involved, requires further analyses.

## The Secyeg Interaction Network

**FIGURE 9:**
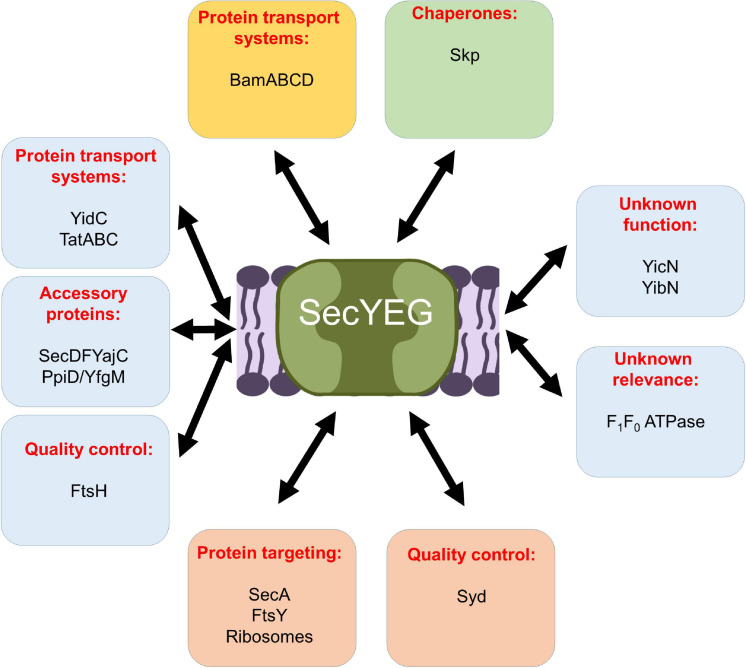
Schematic view on the protein interaction network of the *E. coli* SecYEG complex. Interactions within the inner membrane are shown in blue boxes, those that take place at the cytosolic phase of the inner membrane in orange boxes, those at the periplasmic side of the inner membrane in a green box, and those with the outer membrane are boxed in yellow. For details see text.

The Sec translocon in bacteria and eukaryotes is organized as a highly modular protein complex and multiple different entities have been structurally and biochemically characterized (Zimmer et al., 2008; Boy and Koch, 2009; Frauenfeld et al., 2011; Denks et al., 2014; Komar et al., 2016; Kater et al., 2019). The *E. coli* SecYEG translocon was found to exist as a functional monomer (Menetret et al., 2007; Kedrov et al., 2011; Park and Rapoport, 2012) and as a dimer stabilized by cardiolipin (Gold et al., 2010). SecYEG was furthermore found in heterotetrameric complexes with SecA (Zimmer et al., 2008) or YidC (Boy and Koch, 2009; Sachelaru et al., 2017), and as heterohexameric complexes with SecDFYajC (Duong and Wickner, 1997) or FtsY-SRP-RNCs (Jomaa et al., 2017). Finally, a heteroheptameric SecYEG-SecDFYajC-YidC complex was characterized and referred to as Holo-translocon (HTL) (Schulze et al., 2014; Komar et al., 2016). Several additional partner proteins have been identified, like the YfgM-PpiD chaperone complex (Antonoaea et al., 2008; Götzke et al., 2014; Sachelaru et al., 2014; Furst et al., 2018; Jauss et al., 2019), or the cytosolic protein Syd, which is suggested to serve together with the protease FtsH in quality control of the Sec translocon (Akiyama et al., 1996; Dalal et al., 2009; Table 1 and Figure 9). Non-proteinaceous partners are equally important for SecYEG function, like anionic phospholipids and cardiolipin (Prabudiansyah et al., 2015; Collinson, 2019; Bogdanov et al., 2020; Ryabichko et al., 2020) or the glycolipid MPiase, which was shown to support protein transport via the SecYEG translocon (Moser et al., 2013; Nishiyama and Shimamoto, 2014). The highly dynamic equilibrium between different SecYEG assemblies likely allows the SecYEG complex to adapt to a wide variety of different substrates and to different physiological conditions.

**TABLE 1:** Interaction partners of the SecYEG translocon.

Protein/protein complex	Function	Method of identification	References
**Outer membrane**			
BAM complex	Folding and insertion of OMPs into the OM	Site-directed cross-linking, EM, pull-down, protein modeling	Wang et al., 2016; Alvira et al., 2020; Jin, 2020
Skp	Periplasmic chaperone	Cross-linking, translocation intermediates	Schäfer et al., 1999; Jauss et al., 2019
**Inner membrane**			
F_1_F_0_-ATPase	ATP synthesis coupled proton transport	Native MS, peptidiscs	Chorev et al., 2018; Young et al., 2020
Tat complex	Twin-arginine translocation system	*In vitro* transport studies, qAP/MS	Keller et al., 2012; Tooke et al., 2017; Jauss et al., 2019
YajG	Putative lipoprotein	SEC-PCP-SILAC	Carlson et al., 2019
YibN	Putative sulfurtransferase	qAP/MS; SEC-PCP-SILAC	Carlson et al., 2019; Jauss et al., 2019
YicN	Unknown function	qAP/MS; His-tagged peptidiscs	Carlson et al., 2019; Jauss et al., 2019
YidD	Putative membrane protein insertion efficiency factor	*In vitro* transport/cross-linking	Yu et al., 2011
FtsH	Protease, regulated by the FtsH inhibitor YccA	Co-purification; qAP/MS	Akiyama et al., 1996; van Stelten et al., 2009; Jauss et al., 2019
**Cytoplasm**			
DnaK/DnaJ	Hsp70/Hsp40 chaperone	qAP/MS	Stenberg et al., 2005; Wickstrom et al., 2011b; Castanie-Cornet et al., 2014; Jauss et al., 2019
GroEL	Hsp60 chaperone	Suppressor screen	Danese et al., 1995; Castanie-Cornet et al., 2014
Syd	Membrane associated regulator of SecY function	Suppressor screen, MS	Shimoike et al., 1995; Matsuo et al., 1998; Dalal et al., 2009; Zhang et al., 2012

*Listed are potential interactors of the SecYEG translocon, their localization in the cell, their putative function based on gene ontology (GO) assignments (http://geneontology.org/) and the method that was used for identifying these interactors. Only those interacting proteins are listed for which a detailed characterization of the interaction with the SecYEG complex is still missing. qAP/MS, quantitative affinity purification-mass spectrometry; EM, electron microscopy; SEC-PCP-SILAC, size exclusion chromatography-protein correlation profiling-Stable isotope labeling of amino acids in cell culture.*

### YidC

**FIGURE 10:**
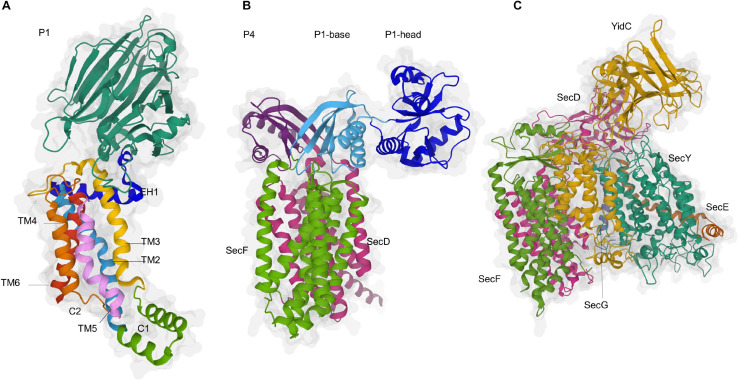
Structures of YidC, SecDF and a model of the holo-translocon. **(A)** Structure of YidC from *E. coli* (PDB 6AL2). The conserved transmembrane domains (TMs) 2 to 6 of YidC are indicated (TM2, light blue; TM3, yellow; TM4, orange; TM5, light pink; TM6, red), while the structure of TM1 is still unknown. The short amphipathic helix EH1 is depicted in dark blue, the periplasmic loop P1 in dark green and the cytoplasmic loop C1 in light green. **(B)** Structure of the SecDF complex from *Thermus thermophilus* (PDB 5YHF). SecDF consists of 12 TMs, six each in SecD (TM1-6, pink) and SecF (TM7-12, green), and three periplasmic domains, termed P1-head (dark blue), P1-base (light blue) and P4 (yellow). **(C)** Modell of the holo-translocon based on the cryo-EM structure from *E. coli* (PDB 5MG3). SecY is shown in green, SecE in orange and SecG in blue, its ancillary subunits SecD in pink, SecF in green and YidC in yellow.

YidC is an inner membrane protein with six TMs in *E. coli* and a N_*in*_-C_*in*_-topology (Figure 10A). It belongs to a conserved group of proteins with homologues in mitochondria, chloroplasts, the endoplasmic reticulum and archaea (Borowska et al., 2015; Anghel et al., 2017; Kuhn and Kiefer, 2017; McDowell et al., 2021). Although YidC can act as SecYEG-independent insertase for some membrane proteins (Samuelson et al., 2000; Luirink et al., 2001; Serek et al., 2004; Welte et al., 2012), it also associates with the SecYEG complex (Scotti et al., 2000; Nouwen and Driessen, 2002; Li et al., 2013; Sachelaru et al., 2015, 2017).

The conserved TMs 2 to 6 of YidC are organized as a globular helix bundle that forms a hydrophilic groove within the membrane, while the structure and position of TM1 is unknown (Kumazaki et al., 2014a, b; Figure 10A). The hydrophilic groove is blocked on the periplasmic side of the membrane by the short amphipathic EH1 helix, which is oriented in parallel to the membrane surface. The EH1 helix is part of the large P1-loop that connects TM1 and TM2 on the periplasmic side (Saaf et al., 1998; Oliver and Paetzel, 2008; Ravaud et al., 2008). On the cytosolic side of TM2, the C1-loop forms a helical coiled-coil domain that is essential for YidC function (Geng et al., 2015). The hydrophilic groove likely faces the TM domains of SecY and cross-link data demonstrate that TM1, TM3 and TM5 of YidC are in close contact to the lateral gate of SecY (Sachelaru et al., 2015; Petriman et al., 2018). YidC can even enter the SecY channel (Sachelaru et al., 2017) and this is achieved via the flexible TM1 and the P1-loop that reaches deep into the periplasmic cavity of SecY, where it makes contact to the plug domain of SecY (Jauss et al., 2019). TM1 was also found in contact with SecG, supporting its intrinsic flexibility (Petriman et al., 2018). Further contacts between YidC and SecY were observed for the C1-loop, while the P1-loop also contacts SecG, SecE and SecF. The C1-loop also provides the docking site for FtsY and is essential for the insertase function of YidC (Geng et al., 2015), but SecY-YidC contacts are maintained even in the absence of the C1-loop (Petriman et al., 2018). Crystallization and molecular dynamics simulations demonstrate that the C2 loop linking TM4 and TM5 is highly flexible (Tanaka et al., 2018). Together with the C-terminus of YidC, the C2-loop provides the ribosome binding site of YidC (Geng et al., 2015) and shields the hydrophilic groove on the cytosolic side (Tanaka et al., 2018). The intimate contact between the hydrophilic groove of YidC and the lateral gate of SecY provides further support for the concept that TMs leaving the SecY channel are first bound by YidC before they are released into the lipid phase (Beck et al., 2001; Houben et al., 2002). TMs exit the SecY channel in most cases sequentially (Serdiuk et al., 2019) and the hydrophilic groove of YidC probably reduces the hydrophobicity of the adjacent lateral gate of SecY and therefore further stimulates the release of the TMs into the inner membrane by a greasy slide. The amphipathic helix EH1 could act as a mechanical switch, tilting TM3 and supporting substrate release (Dalbey et al., 2017; He et al., 2020).

### The SecDFYajC Complex

The inner membrane proteins SecD, SecF and YajC form a stable complex (Pogliano and Beckwith, 1994a, b) and were shown to interact with SecYEG and YidC (Duong and Wickner, 1997; Nouwen and Driessen, 2002). Depletion of SecDF causes cold sensitivity and the accumulation of precursor proteins in the cytosol, supporting their role in stimulating protein translocation across the membrane (Pogliano and Beckwith, 1994a). SecD mutants also lead to elevated levels of SecA (Rollo and Oliver, 1988), which is a typical sign of impaired protein translocation (Ito et al., 2018).

The crystal structure of the SecDF complex shows 12 TMs, six each in SecD and SecF, and three periplasmic domains, termed P1-head, P1-base and P4 (Tsukazaki et al., 2011; Figure 10B). The P1-head can undergo a large rotation, resulting in two distinct conformations, the F- and I-form. An amphiphilic cavity within the P1-head was proposed to bind precursor proteins (Furukawa et al., 2017, 2018). As protein translocation is strongly dependent on the PMF (Driessen and Wickner, 1991; Mori and Ito, 2003; Corey et al., 2018; Knyazev et al., 2018), PMF-driven conformational changes of the P1-head could help to pull substrates out of the SecYEG channel (Tsukazaki et al., 2011; Tsukazaki, 2018). This is in line with the assumption that the SecDF complex is necessary at a later stage of protein translocation (Pogliano and Beckwith, 1994a; Tsukazaki, 2018). The predicted low abundance of the SecDFYajC complex in *E. coli* (Pogliano and Beckwith, 1994a, b) suggests that such a pulling is only required for particular substrates or that other proteins execute a similar function, e.g., the YfgM-PpiD complex that also associates with the SecYEG translocon (Götzke et al., 2014; Sachelaru et al., 2014; Jauss et al., 2019).

SecF interacts with the P1-loop of YidC and the non-conserved residues 215–265 in the P1-loop are sufficient for SecF interaction (Xie et al., 2006), but these residues are not required for YidC function (Jiang et al., 2003). The phenotype of a *secDF* depletion strain can be rescued by YidC-overproduction, further supporting a cooperation between SecDF and YidC (Nouwen and Driessen, 2002; Li et al., 2013). The SecDF complex likely stabilizes the SecYEG-YidC interaction (Nouwen and Driessen, 2002; Tsukazaki, 2018), although the SecYEG-YidC interaction is also observed in the absence of the SecDFYajC complex (Boy and Koch, 2009; Sachelaru et al., 2015). Finally, SecDF might also play a role in efficient maturation and folding of OMPs (Alvira et al., 2020) and it was proposed that SecDF is part of an inter-membrane trafficking machinery that connects transport processes across the inner membrane with those at the outer membrane (Alvira et al., 2020) (see below).

### The Holo-Translocon

The existence of a HTL was first shown after co-expression and purification of its seven constituents (Schulze et al., 2014). The HTL comprises the core SecYEG translocon and its ancillary subunits SecDFYajC and YidC, forming a heteroheptameric complex (Schulze et al., 2014; Botte et al., 2016; Komar et al., 2016). The periplasmic domains of SecDF and YidC are localized on top of the SecY channel and are suggested to interact with emerging substrates, potentially preventing their backsliding (Botte et al., 2016; Figure 10C). The seven subunits of the HTL are arranged around a central lipid-filled chamber, which might provide a flexible and protected environment for TMs to fold, to acquire their final topology and to assemble (Goder et al., 1999; Dowhan et al., 2019; Martin et al., 2019). The presence of the lipid chamber could also promote the assembly of membrane protein complexes, a function that was assigned to YidC when in complex with SecYEG (Wagner et al., 2008). This concept would attribute the HTL a particular role during membrane protein insertion and indeed *in vitro* studies showed that the HTL was more efficient in protein insertion and less effective in SecA-dependent protein secretion than the SecYEG complex (Schulze et al., 2014). However, in these studies the HTL also increased the insertion of proteins that were classified as SecY-independent, like the phage protein Pf3 or subunit c of the F_1_F_0_ ATPase (Serek et al., 2004; van der Laan et al., 2004). The abundance of the HTL in the *E. coli* membrane is not entirely clear. Initial estimations suggested that the SecDF complex is present in only 30–100 copies per cell and thus about 10 times less abundant than SecYEG (Pogliano and Beckwith, 1994a, b). In contrast, ribosome profiling data indicated a 4:1 SecYEG:SecDF ratio (Li et al., 2014) and a recent proteomics study even proposed a 1:1 ratio (Schmidt et al., 2016). Considering that the HTL is only one of several SecYEG assemblies, it is important to emphasize that these absolute numbers would only predict the number of theoretically possible HTLs, but not the real number in the *E. coli* membrane.

### The Interaction of the SecYEG Complex With Periplasmic Chaperones and the Outer Membrane

The interaction of the SecYEG complex with periplasmic chaperones was first shown for Skp and it was suggested that Skp could facilitate substrate release from the SecY channel (Schäfer et al., 1999; Harms et al., 2001; Figure 9). A similar function was also proposed for the membrane-anchored periplasmic chaperone PpiD, which was found to contact a secretory protein exiting SecY (Antonoaea et al., 2008). PpiD forms a complex with YfgM, which contains like PpiD a single TM and a large periplasmic domain (Maddalo et al., 2011; Götzke et al., 2014). YfgM was also found as contact partner of SecYEG and the PpiD-YfgM complex was suggested to mediate substrate transfer from the SecYEG complex to other periplasmic chaperones, like SurA, Skp, or DegP (Götzke et al., 2014; Furst et al., 2018). PpiD contacts the lateral gate of SecY (Sachelaru et al., 2014) and its periplasmic domain deeply inserts into the periplasmic cavity of the SecY channel (Jauss et al., 2019). When the plug domain of SecY is deleted, the interaction between SecYEG and PpiD is enhanced both at the lateral gate as well as in the channel interior which suggests that channel opening controls the SecY-PpiD contact. These SecY-PpiD contacts as revealed by site-directed *in vivo* cross-linking are basically identical to the detected SecY-YidC contacts, which indicates that SecY can either interact with YidC or PpiD. However, PpiD and YidC show non-competitive binding to the SecYEG translocon *in vivo* (Jauss et al., 2019), pointing to the possible presence of two distinct SecYEG populations. This is also supported by Blue-Native PAGE analyses, which found SecYEG either in contact with YidC or PpiD/YfgM (Götzke et al., 2014) and by data showing that the SecY-PpiD contact is lost when SecY is engaged in inserting a membrane protein (Sachelaru et al., 2014). PpiD contains an inactive prolyl-isomerase domain in its periplasmic loop (Weininger et al., 2010) and does not seem to execute any pulling force on SecY substrates (Jauss et al., 2019). Still it improves translocation efficiency and the release of newly translocated substrates into the periplasm, possibly by preventing their backsliding into the periplasmic cavity of SecY (Furst et al., 2018). PpiD was also found to cross-link to the periplasmic chaperone SurA, providing further evidence for a role of PpiD in connecting the translocation machinery to the periplasmic folding machinery (Wang et al., 2016).

After their translocation across the inner membrane, β-barrel OMPs have to be inserted into the outer membrane (Bos et al., 2007; Konovalova et al., 2017). The β-barrel assembly machinery, the BAM complex, is localized in the outer membrane (OM) and facilitates the folding and insertion of OMPs into the OM (Ranava et al., 2018; Ricci and Silhavy, 2019). The complex has a molecular mass of around 203 kDa and comprises the core protein BamA and the four additional lipoprotein subunits BamBCDE (Noinaj et al., 2017; Figure 1). BamA contains a β-barrel domain and five polypeptide-transport-associated (POTRA) domains protruding into the periplasm. Even though only BamA and BamD are essential *in vivo*, all five subunits are necessary for unrestrained function of the complex (Iadanza et al., 2016).

The passage of OMPs from the SecYEG translocon to the BAM complex has been analyzed in multiple studies (reviewed in (Ricci and Silhavy, 2019). A direct interaction between the SecYEG translocon and the BAM complex was first suggested when a supercomplex consisting of BamA, BamB, SurA, PpiD, SecY, SecE, and SecA was found by native gel electrophoresis (Wang et al., 2016). Furthermore, cross-links between the periplasmic chaperone SurA and BamA consolidated the idea that translocation of OMPs across the inner membrane, passage through the periplasm and the insertion into the OM could be physically linked (Wang et al., 2016). BamA was furthermore found to co-purify with the Sec translocon (Jauss et al., 2019) and interactions between SecY and BamACD were identified in a peptidisc approach combined with affinity purification/mass-spectrometry (Carlson et al., 2019). The existence of connecting structures between the inner and outer membranes (so called Bayer’s patches) that could aid the biogenesis of OMPs were first postulated by Bayer (1968). However, they were controversially discussed since their discovery, although some biochemical evidence pointed to the existence of contact points between the outer and inner membrane (Ishidate et al., 1986; Kellenberger, 1990; Malinverni and Silhavy, 2011). This was recently verified by showing the interaction of the HTL with the BAM complex. This transient contact was shown to be conferred by the periplasmic loops of SecDF, YidC, and the BAM complex (Alvira et al., 2020). The periplasmic domain of SecD has multiple contact sites with BamBCD, while YidC interacts with BamABCD. Furthermore, there might be a potential interaction between YajC and BAM (Carlson et al., 2019). In contrast, the SecYEG complex alone is not able to directly interact with the BAM complex, probably due to the lack of large periplasmic domains. The HTL-BAM complex is further stabilized by cardiolipin (Alvira et al., 2020), which was already shown to be important for SecYEG complex stability (Gold et al., 2010; Ryabichko et al., 2020). A yet unsolved question is how OMPs are transported to and inserted into the OM without any apparent energy source due to the lack of ATP in the periplasm and the absence of an ion gradient across the outer membrane (Konovalova et al., 2017). The interaction between the HTL and BAM could facilitate the energetic coupling of inner membrane with outer membrane transport. Once OMP precursors are translocated across the SecYEG complex and the signal sequence is cleaved, the mature but yet unfolded protein is bound by periplasmic chaperons, such as PpiD (Antonoaea et al., 2008) and is then recognized by the BAM complex, forming a trans-periplasmic supercomplex with SecDF as potential energy supplier (Carlson et al., 2019; Alvira et al., 2020).

### Further Contacts of the SecYEG Complex

Functional and proteomic studies have identified several additional proteins as potential contact partners of the SecYEG complex (Kuhn et al., 2011; Carlson et al., 2019; Jauss et al., 2019), although the functional relevance of some of these interactions require further analyses (Table 1 and Figure 9).

The cytosolic protein Syd was shown to stabilize overexpressed SecY in *E. coli* (Shimoike et al., 1995) and to prevent access of SecA to an altered SecYEG translocon (Matsuo et al., 1998). Syd is suggested to bind to the C4 and C5 loops of SecY (Dalal et al., 2009), which are also part of the SecA binding site (Mori and Ito, 2006; Kuhn et al., 2011) and it appears that binding of SecA and Syd to SecY is mutually exclusive (Dalal et al., 2009). The SecY-Syd interaction could provide a quality control system for the correct assembly of the SecYEG complex, probably in conjunction with the essential zinc-metalloprotease FtsH (Kihara et al., 1995; Ito and Akiyama, 2005).

A cooperation between the SecYEG translocon and the Tat transport system for folded proteins (Kudva et al., 2013) was observed in *Streptomyces coelicolor* (Keller et al., 2012). Here, the first two TMs of the Rieske iron-sulfur protein are inserted via the SecYEG translocon, while TM3 is dependent on the Tat machinery. The dual requirement for the Sec- and Tat-machinery appears to be common for membrane proteins that contain globular, co-factor containing extracytoplasmic domains (Tooke et al., 2017), which are abundant in both Gram-positive and Gram-negative bacteria. TatA was also found co-purifying with the SecYEG translocon in *E. coli*, supporting the concept of a widespread cooperation between the Sec and Tat transport systems (Jauss et al., 2019).

The F_1_F_0_-ATPase was also shown to interact with the SecYEG complex (Chorev et al., 2018) and subunit *b* of F_1_F_0_-ATPase was enriched in a peptidisc approach (Young et al., 2020). The interaction of the protein translocation machinery with components of the respiratory chain has been extensively studied in the mitochondrial inner membrane (Pfanner et al., 2019), but the physiological importance of these interactions in the bacterial membrane requires further analyses.

YibN and YicN are two single-spanning membrane proteins of approx. 15 kDa that co-purify with SecYEG (Jauss et al., 2019; Young et al., 2020), but their functions have not been elucidated. A possible role of YibN in protein transport is supported by the observation that YibN is up-regulated when YidC is depleted (Wickstrom et al., 2011b) and in particular enriched when the SecYEG translocon is purified from *secDF*-depleted *E. coli* strains (Young et al., 2020). Nevertheless, the exact role of YibN/YicN in the translocation machinery and how they interact with the Sec translocon has still to be examined.

## Connecting Protein Transport to the Proteostasis Network

**FIGURE 11:**
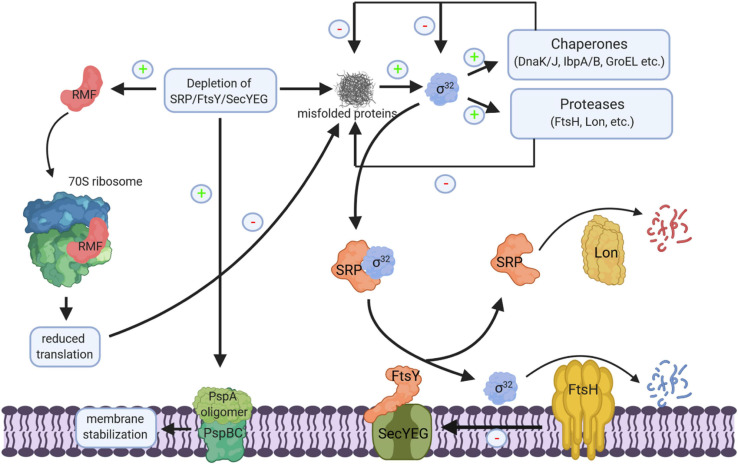
Cellular response to impaired protein transport. Depletion of SRP/FtsY or SecYEG induce a multifaceted response. This includes membrane stabilization via the induction of the phage-shock response (PspABC complex), the inhibition of translation via the induction of the ribosome-modulation factor (RMF) upon FtsY depletion and the induction of the σ^32^-response via the accumulation of misfolded proteins. Increased chaperone and protease production reduce the cellular concentration of misfolded proteins and provide a negative feedback loop for declining the σ^32^ response. Chaperones inhibit σ^32^ directly and the membrane bound protease FtsH degrades σ^32^. Membrane targeting of σ^32^ for degradation by FtsH is dependent on SRP/FtsY and SecYEG. Thus, upon SRP/FtsY or SecYEG depletion/saturation, elevated σ^32^ levels persist. FtsH also degrades misfolded/aggregated membrane proteins and SecY that is not in complex with its partner protein SecE. Ffh, the protein subunit of SRP is also a substrate of the Lon protease; in particular when Ffh is in excess over the 4.5S RNA, the RNA subunit of the bacterial SRP. “+” indicates increased production, “–” indicates reduced production, inhibition or degradation.

The SecYEG translocon, SecA, SRP, and FtsY are essential for cell viability, but conditional depletion strains have been generated for some of the respective genes and were analyzed for transcriptomic or proteomic responses. The depletion of SRP induces the σ^32^-response and leads to an up-regulation of several chaperones and proteases, like DnaK, GroEL, GroES, ClpB, IbpA, and FtsH (Bernstein and Hyndman, 2001; Wickstrom et al., 2011a; Figure 11). It furthermore induces the phage-shock protein A (PspA), which is generally associated with inner membrane damage (Manganelli and Gennaro, 2017). However, it does not lead to increased levels of stress-induced periplasmic proteins, like DegP or Skp (Wickstrom et al., 2011a), suggesting that the σ^E^-dependent cell envelope stress response is not induced (Hews et al., 2019). This is rather surprising, because the insertion of SecY is dependent on the SRP/FtsY pathway (Koch and Muller, 2000) and SRP depletion should reduce the levels of SecY, which subsequently should impair the translocation of OMPs (Kudva et al., 2013). On the other hand, by promotor fusion experiments it was shown that impaired SecY activity is not strictly linked to the induction of the cell envelope stress response (Shimohata et al., 2007). It appears likely that the σ^E^-dependent cell envelope stress response is only induced upon prolonged SRP-depletion or when SecYEG-dependent transport largely ceased. The up-regulation of chaperones and proteases is also observed in a conditional FtsY-depletion strain. However, FtsY-depletion additionally induced ribosome-inactivation via the ribosome-modulation factor (RMF) (Bürk et al., 2009). An up-regulation of chaperones/proteases and down-regulation of translation is also observed in eukaryotic cells upon SRP depletion (Mutka and Walter, 2001). Importantly, the depletion of the SRP pathway in either bacteria or eukaryotic cells does not cause a rapid decline in the membrane proteome (Ulbrandt et al., 1997; Wickstrom et al., 2011a; Costa et al., 2018). A possible explanation for this conundrum is the intrinsic affinity of ribosomes for the SecYEG complex (Prinz et al., 2000) and the presence of alternative targeting systems in eukaryotes (Ast et al., 2013).

The cellular concentration of SRP is controlled by the Lon protease, which is induced upon stress conditions. However, Lon-dependent degradation of Ffh primarily occurs when the Ffh levels exceed the concentration of the 4.5S RNA (Sauerbrei et al., 2020) and it is unclear whether Lon also reduces the Ffh levels upon stress conditions. FtsY is encoded in the *ftsYEX* operon, upstream of the heat-shock sigma factor σ^32^ (Gill and Salmond, 1987, 1990; Weinreich et al., 1994), however, they seem not to be transcriptionally coupled (Gómez-Eichelmann and Helmstetter, 1999). FtsE and FtsX are involved in the control of peptidoglycan hydrolase activity and important for cell division (Pichoff et al., 2019), explaining the filamentous phenotype of *ftsYEX* mutations (Luirink et al., 1994). FtsY levels have been shown to increase at low temperature (Liu et al., 2016; Zhong and Zhao, 2019) and FtsY is subject to a proteolytic event, which degrades its N-terminal membrane targeting sequence (Weiche et al., 2008). However, the responsible protease and the physiological significance of this degradation are still unknown.

Mutants lacking SecB or depleted for SecA also show an up-regulation of the σ^32^-response due to the accumulation of secretory protein precursors in cytoplasm (Wild et al., 1992, 1993, 1996). SecB-deficient strains also show impaired growth on rich medium (Kumamoto and Beckwith, 1985; Wild et al., 1993), however, this is likely caused by a polar effect of the *secB* deletion on the downstream *gpsA* gene, which is involved in phospholipid biosynthesis (Shimizu et al., 1997).

The σ^32^-response and the formation of cytosolic aggregates containing many ribosomal proteins is also induced upon SecYE depletion (Wild et al., 1992, 1993, 1996; Baars et al., 2008). However, in comparison to SRP depletion, SecYE depletion has a more drastic effect on the steady-state levels of inner membrane proteins and secretory proteins (Baars et al., 2008). SecYE-depletion primarily reduces the levels of multi-spanning membrane proteins and the levels of membrane proteins with large periplasmic domains. Intriguingly, these membrane proteins cannot engage YidC as second integration site for membrane proteins (Samuelson et al., 2000; Serek et al., 2004) and are therefore strictly dependent on SecYEG. The levels of single spanning and short membrane proteins are less impaired by SecYE-depletion, because they can use YidC as alternative integration site when SecYEG is depleted. This is also in line with the observation that the SRP pathway can target both SecYEG and YidC (Welte et al., 2012; Petriman et al., 2018).

The σ^32^-response in *E. coli* is regulated by two feedback loops. Free chaperones, like DnaK or GroEL bind and inactivate σ^32^, while the inner membrane protease FtsH degrades σ^32^. It was recently shown that membrane targeting of σ^32^ is dependent on SRP, FtsY, and SecY (Lim et al., 2013; Miyazaki et al., 2016; Figure 11). Thus, depletion of SRP/FtsY increases the stability of σ^32^ by reducing its degradation via FtsH. This allows for increased chaperone and protease production when the SRP pathway or the SecYEG translocon are saturated and links protein transport directly to the proteostasis network.

The levels of SecY and SecE in *E. coli* are slightly higher on rich media compared to minimal media and are reduced in stationary phase (Yang et al., 2013; Crane and Randall, 2017). Thus, the expression of *secY* and *secE* seem to mimic the expression of house-keeping genes. A similar observation was made for *secDF* expression in *S. coelicolor* (Zhou et al., 2014). This is different for SecA; here an intriguing mechanism has been identified that allows *E. coli* to tailor SecA-levels to reduced translocation activity of the SecYEG translocon (Ito et al., 2010; Ito and Chiba, 2013). This was first recognized by studies showing that partial inactivation of SecYEG-dependent translocation by *secY* mutations or by adding the SecA-inhibitor sodium azide, led to an up-regulation of SecA (Oliver and Beckwith, 1982; Rollo and Oliver, 1988). This regulation is achieved by the product of the upstream *secM* gene, which is co-transcribed with *secA*. Both genes are separated on the mRNA by a stem-loop- like sequence that overlaps with the Shine-Dalgarno sequence of *secA*. SecM (*secretion monitor*) is a signal-sequence containing polypeptide that is translocated into the periplasm, where it is rapidly degraded. A particular feature of SecM is the presence of a stalling sequence at its C-terminus, which causes a transient translation arrest that is released during translocation. However, when translocation is compromised, translational arrest persists and the formation of the stem-loop is blocked, allowing the ribosome unhindered access to the Shine-Dalgarno sequence of the *secA* gene and increases the production of SecA (Ito et al., 2018). The use of monitoring substrates for adjusting the protein transport capacity has also been shown in *Vibrio alginolyticus*, where the substrate VemP controls the switch between a sodium-coupled SecDF2 complex and a proton-coupled SecDF1 complex in low Na^+^ environments (Ishii et al., 2015; Miyazaki et al., 2020). Similar systems are also active in Gram-positive bacteria, like *B. subtilis*. Here, the monitoring substrate MifM controls the expression of the alternative YidC2 when YidC1 is compromised (Chiba et al., 2011; Chiba and Ito, 2012, 2015).

Besides the minor growth-phase dependent regulation of SecY and SecE as described above, entries in the *E. coli* gene expression database do not reveal a strong transcriptional regulation of the respective genes in response to different growth or stress conditions (GenExpDB^3^). This is also validated by a proteomic approach, which demonstrated comparable levels of SecY, SecE and SecG over the entire growth phase of *E. coli* (Soufi et al., 2015). This is rather surprising, because *secY* is encoded in the *spc* operon together with genes for several ribosomal proteins (Lindahl et al., 1990; Ikegami et al., 2005). These genes are significantly down-regulated during stationary phase or when cells encounter stress conditions (Coenye and Vandamme, 2005; Ikegami et al., 2005; Starosta et al., 2014). The *spc* operon is under control of the *rplN* promotor and binding of RNA-polymerase is inhibited when cells enter stationary phase by the transcription factor DksA and the alarmone ppGpp, a hyper-phosphorylated guanosine derivative (Lemke et al., 2011; Haas et al., 2020). Thus, *secY* expression is obviously disconnected from the regulation of the other genes within the *spc* operon, probably by the presence of an internal promotor.

**FIGURE 12:**
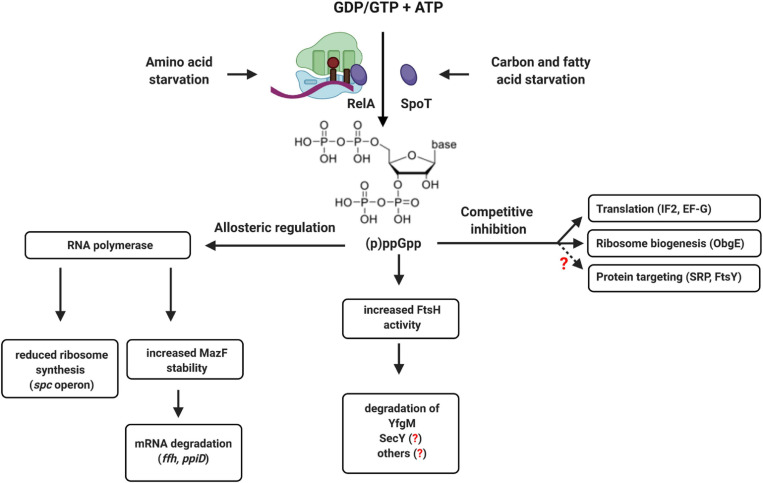
(p)ppGpp-dependent regulation of translation and protein transport in bacteria. The alarmones ppGpp and pppGpp are synthesized upon amino acid starvation by the ribosome-associated protein RelA or by the cytosolic protein SpoT upon carbon or fatty acid starvation. Allosteric regulation of RNA polymerase by (p)ppGpp reduces ribosome biogenesis and increases the stability of the ribonuclease MazF, which degrades multiple mRNAs. This includes the mRNA encoding for Ffh, the protein component of the bacterial SRP, or the *ppiD* mRNA, encoding for an accessory subunit of the SecYEG translocon. (p)ppGpp also increases the activity of FtsH, which can degrade SecY and YfgM. YfgM forms a complex with PpiD that associates with the SecYEG translocon. Whether SecY is specifically degraded by FtsH upon (p)ppGpp accumulation is not shown yet. (p)ppGpp also acts as competitive inhibitor of GTP-binding proteins like translation factors (IF2 and EF-G) or ribosome biogenesis proteins (ObgE). This leads to reduced ribosome biogenesis and reduced translation upon stress. Although not yet experimentally shown, it appears likely that increasing (p)ppGpp concentrations also inhibit the two GTPases SRP and FtsY, which would fine-tune the protein targeting machinery to the reduced translation rates.

In *E. coli*, the levels of the two alarmones ppGpp and pppGpp are mainly controlled by the activity of two enzymes, RelA and SpoT (Atkinson et al., 2011; Potrykus and Cashel, 2018; Pausch et al., 2020). RelA primarily responds to stalled ribosomes upon amino acid starvation (Starosta et al., 2014; Steinchen et al., 2020), while SpoT activity increases upon fatty acid or carbon starvation (Battesti and Bouveret, 2009; Figure 12). High levels of (p)ppGpp induce a process called stringent response that is associated with a significant re-programming of cellular activities (Bennison et al., 2019; Irving et al., 2020). The (p)ppGpp levels raise from approx. 40 μM during exponential phase up to approx. 1 mM at the transition into stationary phase or upon amino acid starvation (Varik et al., 2017; Haas et al., 2020; Steinchen et al., 2020). Cellular re-programming is induced by two mechanisms: allosteric regulation of target proteins, like RNA polymerase, which leads to reduced expression of the *spc*-operon (Liang et al., 1999; Steinchen et al., 2020), and competitive inhibition of GTP-binding proteins, like the ribosome assembly factor ObgE (Sato et al., 2005; Persky et al., 2009; Feng et al., 2014), the initiation factor IF2 (Diez et al., 2020) or elongation factor EF-G (Mitkevich et al., 2010; Steinchen et al., 2020). As a result, ribosome biogenesis and translation are adjusted to substrate limitation.

Increasing (p)ppGpp concentrations likely also interfere with the activity of the GTPases FtsY and SRP and both proteins were identified as potential targets of (p)ppGpp (Wang B. et al., 2019). This would enable cells to adjust the protein targeting machinery to the reduced protein synthesis rate upon entry into stationary phase or during nutrient limitation. However, the consequences of (p)ppGpp on SRP-dependent protein targeting have not been studied so far. The accumulation of ppGpp also activates the MazEF toxin-antitoxin system (Moll and Engelberg-Kulka, 2012) and the mRNAs of both PpiD and Ffh were identified as potential targets of the riboendonuclease MazF (Sauert et al., 2016). This provides an additional link between stress conditions and the protein targeting and transport machinery that requires further analyses. Bacteria also produce hyper-phosphorylated adenosine derivatives, like (p)ppApp, although less is known about the conditions of synthesis and potential regulatory consequences (Travers, 1978; Bruhn-Olszewska et al., 2018; Ahmad et al., 2019). Still, it is tempting to speculate that by accumulating (p)ppGpp or (p)ppApp, bacteria can adjust protein transport by an allosteric or competitive mechanism, rather than by transcriptional or translational regulation. ppGpp also induces FtsH-dependent degradation of the SecYEG-interacting protein YfgM when cells enter stationary phase (Bittner et al., 2015). This is suggested to relieve the response regulator RcsB, thereby allowing cellular protection by the Rcs phosphorelay system (Lasserre et al., 2006; Wall et al., 2018). However, this would also reduce the levels of the PpiD-YfgM complex and thus impact on the SecYEG interactome under stress conditions. How stress conditions influence the steady-state levels of the protein transport machinery and the dynamic equilibrium between the different SecYEG assemblies is largely a *terra incognita*, but a promising area for future research.

## Inhibitors of Secyeg-Dependent Protein Transport

**FIGURE 13:**
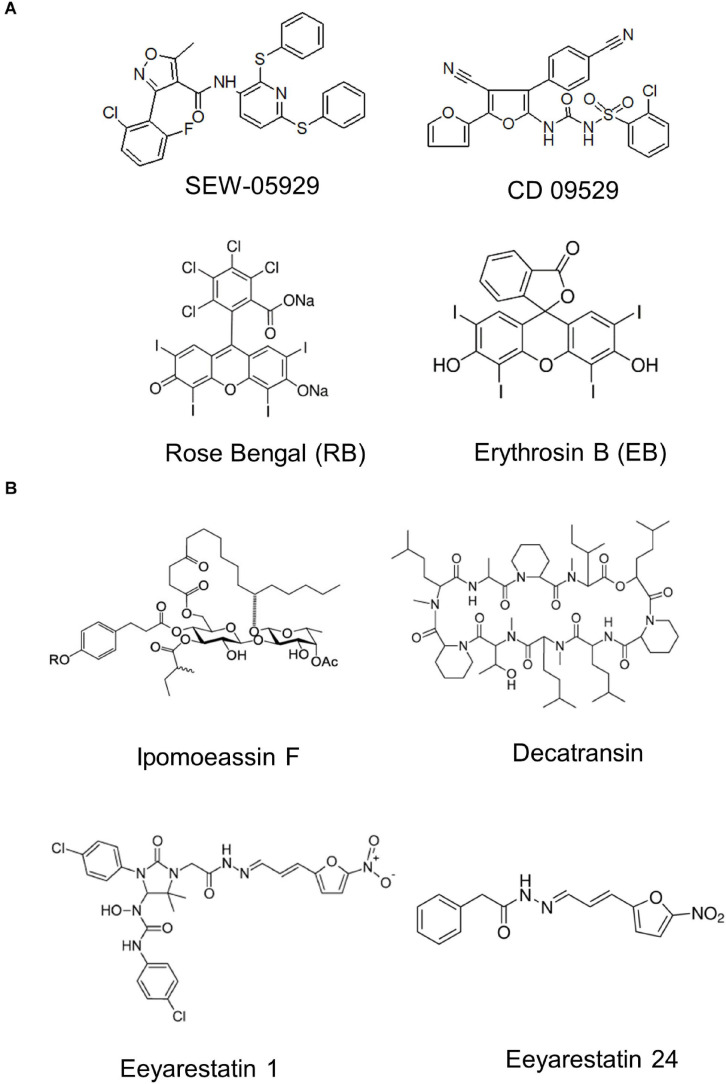
Inhibitors of bacterial protein translocation. **(A)** Inhibitors of the ATPase SecA. **(B)** Inhibitors of the SecYEG-translocon. Ipomeassin F, decatransin, eeyarastatin 1 and eeyarastatin 24 also act on the homologous Sec61 complex in eukaryotes. Chemical structures were retrieved from the Sigma Aldrich web resource (https://www.sigmaaldrich.com/) or adapted from (Li et al., 2008; Van Puyenbroeck and Vermeire, 2018; Zong et al., 2019).

The rapid rise of antibiotic resistance is a major problem for treating infections and novel antimicrobial strategies are of crucial importance (Rodríguez-Rojas et al., 2013; Sulaiman and Lam, 2021). Initial studies on exploring the protein transport machinery as potential target were focused on SecA inhibitors, because SecA homologues are absent in metazoans and SecA inhibition would affect most periplasmic and OMPs as well as some inner membrane proteins (Pohlschroder et al., 2005). Azide was the first described inhibitor of SecA (Oliver et al., 1990), but has no medical relevance due to its high toxicity (Chang and Lamm, 2003). Additional small molecule SecA inhibitors with broad-spectrum activity have been developed and include compounds like SEW-05929 and CD 09529, which inhibit the ATPase activity of SecA but are inactive on wild type *E. coli* strains (Li et al., 2008; Figure 13). Further studies identified 4-oxo-5-cyano thiouracils (Chaudhary et al., 2015), Fluorescein analogs (Huang et al., 2012) and triazole-pyrimidine analogs (Cui et al., 2016; Jin et al., 2016) as SecA inhibitors that are active against *E. coli* and *S. aureus* (Rao et al., 2014; De Waelheyns et al., 2015; Van Puyenbroeck and Vermeire, 2018).

The first characterized inhibitors of the Sec complex were synthetic signal peptides that have been shown to inhibit the eukaryotic Sec61 complex (Austen et al., 1984). The mammalian Sec61 complex is also inhibited by lanthanum ions, which stabilize the Sec61 channel in its open state (Erdmann et al., 2009). Components that inhibit both the eukaryotic Sec61 complex and the bacterial SecYEG complex are the glycoresin Ipomoeassin F (IpomF) (Zong et al., 2019; Steinberg et al., 2020), eeyarestatin (Cross et al., 2009; Steenhuis et al., 2021) and decatransin (Junne et al., 2015; Kalies and Römisch, 2015). IpomF was isolated from the morning glory *Ipomea squamosa* and shown to bind most likely near the lateral gate of Sec61α (Zong et al., 2019). IpomF also inhibits SecYEG-dependent transport *in vitro*, but this requires significantly higher concentrations than required for inhibition of Sec61-dependent transport (Zong et al., 2019; Steinberg et al., 2020). IpomF does not prevent the initial contact of substrate proteins with the SecYEG translocon, but rather blocks later stages of translocation (Steinberg et al., 2020).

Eeyarestatin I (ESI) was initially discovered as inhibitor of the retrograde protein transport into the endoplasmic reticulum and then shown to inhibit co-translational protein transport by the Sec61 complex (Cross et al., 2009). ESI does not inhibit growth of *E. coli*, but a smaller variant of ESI, ES24 (Gamayun et al., 2019), is active against *E. coli* and several clinically relevant pathogens (Steenhuis et al., 2021). ES24 likely binds to the cytosolic part of the lateral gate (Gamayun et al., 2019), but the antibacterial activity depends on the presence of the nitroreductases NfsA and NfsB, indicating that a specific reduction step is required to activate ES24 (Steenhuis et al., 2021). Decatransin is a naturally occurring fungal decadepsipeptide that was identified in a cancer drug screen and later shown to inhibit SecYEG/Sec61. Decatransin-resistant mutations mapped to the pore ring and to the plug of the Sec channel, suggesting that decatransin interferes with channel opening (Junne et al., 2015). However, whether these SecA- and SecY-inhibiting compounds also have clinical relevance requires further investigation.

## Conclusion and Outlook

The bacterial SecYEG translocon has been the focus of intense research for decades and served as a paradigm for genetic, biochemical and structural studies on protein transport mechanisms. The progress that has been made from the early genetic screens (Bassford et al., 1991; Beckwith, 2013) to the currently available structures is incredible (Smets et al., 2019; Tanaka and Tsukazaki, 2019). Snap-shots of the SecYEG translocon in contact with its most prominent partner proteins and of the SecYEG translocon in action during translocation or insertion of protein substrates have been attained and provide first insights into how these protein transport channels work. Still, structural information of substrate-engaged larger SecYEG assemblies, like the SecYEG-YidC complex, the SecYEG-PpiD/YfgM complex or the HTL, are needed for understanding how the SecYEG translocon handles the large variety of potential substrates. Equally needed are structures of the SecYEG translocon during the insertion of multi-spanning membrane proteins. It is also evident that the current picture of the SecYEG interactome is incomplete and includes only the most stable and abundant partner proteins. Many transient interactions only emerged upon improved mass spectrometry methods (Carlson et al., 2019; Jauss et al., 2019) and the functional characterization of these transient contacts will be a major challenge for the future. This will be particularly demanding if these contacts are only required for a specific subset of substrates, which are not in the tool box of frequently used model substrates. Analysing the transport of membrane proteins with large soluble domains at the N-terminus (Facey and Kuhn, 2003; Maier et al., 2008; Rawat et al., 2015; Wang S. et al., 2019) or very small membrane proteins, which basically consist of just a single transmembrane domain, has already revealed unexpected targeting and insertion requirements (Steinberg et al., 2018, 2020). Despite the increasing number of proteins interacting with the SecYEG translocon, the number of identified contact sites on SecY is rather low and mainly includes the cytosolic loop 5, the lateral gate and the periplasmic vestibule. This suggests that some proteins either compete for SecY binding, or interact with dedicated sub-populations of the SecYEG translocon and these subpopulations need to be further characterized. Our current view on bacterial protein transport pathways follows a rather strict dissection into multiple separate transport pathways, but recent data suggest that these pathways are intertwined. The best-studied example is of course the SecYEG-YidC interaction (Scotti et al., 2000), where YidC likely helps substrates to exit the SecY channel (Beck et al., 2001; Houben et al., 2002), although YidC can also act as SecYEG-independent insertase (Samuelson et al., 2000; Serek et al., 2004). But there are more examples, like the SecYEG-Tat interaction (Keller et al., 2012) or the SecYEG-Bam interaction (Alvira et al., 2020), and the collaboration between different transport systems needs to be further explored. Finally, it is largely unknown how the protein transport machinery responds to environmental changes or to stress conditions. Considering the multifaceted responses that down-regulate protein synthesis when cell encounter non-favorable conditions, it appears more than likely that similar, but so far unexplored mechanisms, also modulate the protein transport capacity of the cell. Thus, there is still a lot to learn about the SecYEG translocon or, to cite famous Isaac Newton: “What we know is a drop. What we don’t know is an ocean.”

## Author Contributions

JO, RN, AN, PS, and H-GK designed the review, prepared the figures, and wrote the manuscript. All authors contributed to the article and approved the submitted version.

## Conflict of Interest

The authors declare that the research was conducted in the absence of any commercial or financial relationships that could be construed as a potential conflict of interest.

## Footnotes

1 https://www.ncbi.nlm.nih.gov/genome/

2 https://stepdb.eu/

3 https://genexpdb.okstate.edu/databases/genexpdb/
